# A threshold of transmembrane potential is required for mitochondrial dynamic balance mediated by DRP1 and OMA1

**DOI:** 10.1007/s00018-016-2421-9

**Published:** 2016-11-17

**Authors:** Edith Jones, Norma Gaytan, Iraselia Garcia, Alan Herrera, Manuel Ramos, Divya Agarwala, Maahrose Rana, Wendy Innis-Whitehouse, Erin Schuenzel, Robert Gilkerson

**Affiliations:** 1 Department of Biology, The University of Texas Rio Grande Valley, 1201 West University Drive, Edinburg, TX 78539-2999 USA; 2 Department of Biomedical Sciences, The University of Texas Rio Grande Valley, Edinburg, TX 78539-2999 USA; 3 Department of Clinical Laboratory Sciences, The University of Texas Rio Grande Valley, 1201 West University Drive, Edinburg, TX 78539-2999 USA

**Keywords:** Oxidative phosphorylation, mtDNA, S-OPA1, Protonophore, Proteolytic cleavage

## Abstract

As an organellar network, mitochondria dynamically regulate their organization via opposing fusion and fission pathways to maintain bioenergetic homeostasis and contribute to key cellular pathways. This dynamic balance is directly linked to bioenergetic function: loss of transmembrane potential across the inner membrane (Δ*ψ*_m_) disrupts mitochondrial fission/fusion balance, causing fragmentation of the network. However, the level of Δ*ψ*_m_ required for mitochondrial dynamic balance, as well as the relative contributions of fission and fusion pathways, have remained unclear. To explore this, mitochondrial morphology and Δ*ψ*_m_ were examined via confocal imaging and tetramethyl rhodamine ester (TMRE) flow cytometry, respectively, in cultured 143B osteosarcoma cells. When normalized to the TMRE value of untreated 143B cells as 100%, both genetic (mtDNA-depleted ρ^0^) and pharmacological [carbonyl cyanide m-chlorophenyl hydrazone (CCCP)-treated] cell models below 34% TMRE fluorescence were unable to maintain mitochondrial interconnection, correlating with loss of fusion-active long OPA1 isoforms (L-OPA1). Mechanistically, this threshold is maintained by mechanistic coordination of DRP1-mediated fission and OPA1-mediated fusion: cells lacking either DRP1 or the OMA1 metalloprotease were insensitive to loss of Δ*ψ*_m_, instead maintaining an obligately fused morphology. Collectively, these findings demonstrate a mitochondrial ‘tipping point’ threshold mediated by the interaction of Δ*ψ*_m_ with both DRP1 and OMA1; moreover, DRP1 appears to be required for effective OPA1 maintenance and processing, consistent with growing evidence for direct interaction of fission and fusion pathways. These results suggest that Δ*ψ*_m_ below threshold coordinately activates both DRP1-mediated fission and OMA1 cleavage of OPA1, collapsing mitochondrial dynamic balance, with major implications for a range of signaling pathways and cellular life/death events.

**Electronic supplementary material:**

The online version of this article (doi:10.1007/s00018-016-2421-9) contains supplementary material, which is available to authorized users.

## Introduction

Mitochondria have emerged as a highly responsive organellar network that dynamically balances between two states: a collection of individual spherical organelles, or an elaborately interconnected reticular network [1, 2], thus adapting its organization to directly integrate into crucial cellular processes including metabolism, apoptosis, autophagy, and proliferation [3]. Moreover, mitochondrial dynamics are directly linked with bioenergetic function in an integrated structure/function relationship: loss of transmembrane potential (Δ*ψ*_m_), which is critical to oxidative phosphorylation (OxPhos), causes collapse of structural homeostasis, leading to complete fragmentation of the mitochondrial network [4]. Despite this, it has remained unclear what level of bioenergetic function is required for mitochondrial dynamic balance, as well as the contributing mechanisms necessary. Here, our results reveal a ‘tipping point’ threshold of mitochondrial structure/function.

Mitochondrial dynamics employ an elegant balance of fusion and fission pathways, with each process mediated by a distinct set of interacting factors. Fusion is carried out by optic atrophy-1 (OPA1), a dynamin-related GTPase [5] that mediates fusion of the inner membrane [6, 7], while fusion of the outer mitochondrial membrane is accomplished by mitofusins 1 (MFN1) and 2 (MFN2). Fusion of the mitochondrial outer and inner membranes allows individual organelles to exchange components [4] and permits complementation between functional and dysfunctional organelles [8]. The opposing process, fission, is mediated by dynamin-related protein-1 (DRP1) [9], which is recruited to the mitochondrial outer membrane by interacting proteins FIS1 [10, 11] and MFF1 [12] where it forms an oligomeric ring, dividing mitochondria at discrete sites. As these processes are carried out by different sets of interacting factors, inhibition of one pathway causes an increase in the other: inhibition of DRP1-mediated fission causes unopposed mitochondrial fusion [13, 14]. In a unique organellar structure/function relationship, fission/fusion dynamics are increasingly linked to mitochondrial bioenergetics via the transmembrane potential across the inner membrane (Δ*ψ*_m_) [15].

Mitochondrial ATP production is accomplished by the five OxPhos complexes in the mitochondrial inner membrane. Complexes I–IV utilize electron transfer to generate the proton-motive Δ*ψ*_m_ that is used by Complex V, the F_1_F_0_ ATP synthase, to create ATP from ADP and P_i_. These complexes are composed of polypeptide subunits encoded on both chromosomal and mitochondrial DNA (mtDNA). Strikingly, cells with partial or complete loss of Δ*ψ*_m_ are unable to maintain an interconnected, reticular mitochondrial morphology: cells depleted of mtDNA (ρ^0^ cells), as well as cells carrying mtDNA mutations affecting Δ*ψ*_m_, show completely fragmented mitochondrial ultrastructure [16–19], while pharmacological dissipation of Δ*ψ*_m_ via the protonophore CCCP and the ionophore valinomycin also cause fragmentation of the mitochondrial network [4, 20]. While the mitochondria of mtDNA-depleted ρ^0^ cells show a weak ability to fuse and exchange contents via ‘kiss-and-run’ events [21], both ρ^0^ and CCCP-treated cells are unable to maintain fission/fusion balance, with their mitochondria existing instead as a fragmented population of organelles [4, 21]. Despite the importance of Δ*ψ*_m_ to mitochondrial structure/function homeostasis, however, it is unclear i) what level of Δ*ψ*_m_ is required for maintenance of mitochondrial fission/fusion balance, and ii) the relative contribution of the opposing fusion and fission processes to Δ*ψ*_m_-dependent mitochondrial dynamics in the cell.

Fission/fusion dynamics are integral to mitochondrial participation in vital cellular processes: fission is required for stemness [22], mitosis [23, 24], apoptosis [25], and autophagy [26, 27], while fusion is a necessary adaptation to nutrient starvation and increased metabolic demand [3], allowing transmission of Δ*ψ*_m_ along interconnected mitochondria [1]. Moreover, disruption of mitochondrial dynamics and bioenergetics are emerging in prevalent diseases such as heart failure and neurodegenerative disorders [28, 29]. As such, the interaction of Δ*ψ*_m_ and fission/fusion balance is likely to have major impact as an underlying mechanism of prevalent human disease.

In the classical mitochondrial genetic threshold effect, cells can withstand a high mtDNA mutation load (often up to 80–90% mutant mtDNA) and maintain full OxPhos function, but when the overall proportion of mutant mtDNA exceeds a critical threshold, mitochondrial bioenergetic function collapses, leading to a variety of systemic and tissue-specific pathologies [30, 31]. Previously, we showed that heteroplasmic cells (carrying both WT and mutant mtDNAs) above 80% mutation load have completely fragmented mitochondria, while cells below 80% mutation load have effective fission/fusion balance [17], suggesting that a functional threshold of Δ*ψ*_m_ is required to maintain effective fission/fusion balance. Intriguingly, the OPA1 fusion factor undergoes cleavage and inactivation by the OMA1 metalloprotease in response to dissipation of Δ*ψ*_m_, causing mitochondrial fragmentation [32–34]; however, the level of Δ*ψ*_m_ required and the necessity of coordinating OPA1 cleavage with other fission/fusion factors in balancing mitochondrial dynamics and function remains unclear.

To explore this, we employed genetic and pharmacological models of decreased Δ*ψ*_m_ to determine the functional and mechanistic requirements for effective mitochondrial fission/fusion dynamic balance in human cells. Cultured cells grown in high-glucose media obtain ATP primarily via glycolysis, allowing the growth and study of cells with genetic or pharmacological defects in bioenergetics [35]. Using TMRE flow cytometry to monitor Δ*ψ*_m_, our data indicate that cells below 34% of untreated wild-type (WT) TMRE values cannot maintain mitochondrial dynamic balance, with concomitant loss of fusion-active OPA1: this threshold is mediated by DRP1 and OMA1, reflecting a crucial breakpoint of Δ*ψ*_m_ as a determinant of mitochondrial homeostasis, with severe impacts on cell viability and broad relevance to human disease.

## Results

### Cells with low Δ*ψ*_m_ lack reticular mitochondrial organization

**Fig. 1:**
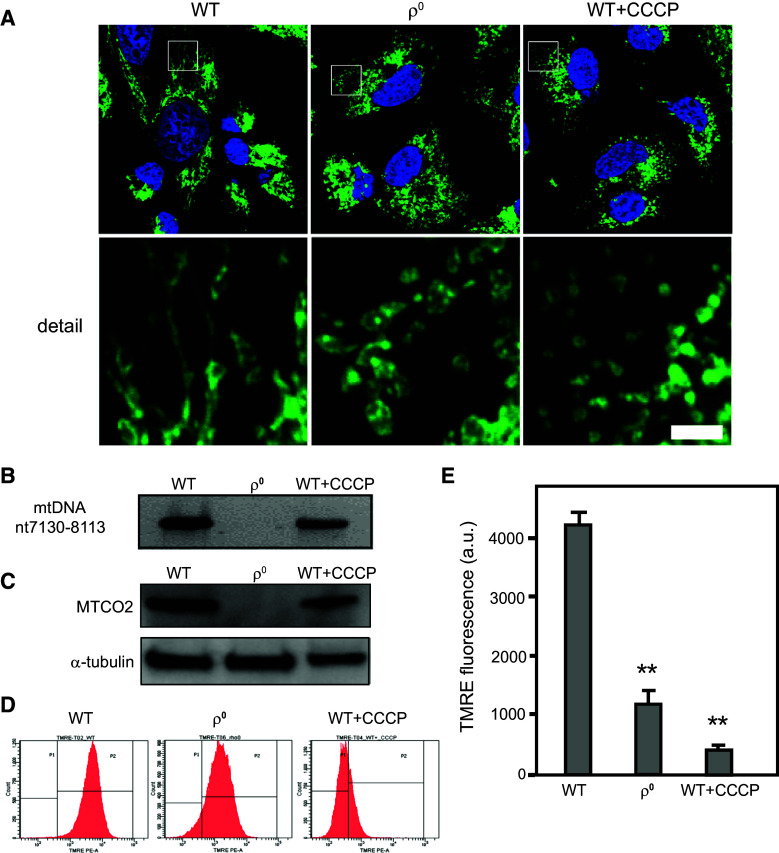
Mitochondrial morphology and bioenergetics in human 143B osteosarcoma cells. **a** Cultured 143B cells visualized by confocal fluorescence microscopy. WT, mtDNA-depleted ρ^0^, and CCCP-treated (10 μM, 1 h) WT cells were immunolabeled for mitochondrial TOM20 (*green*). Nuclei stained with DAPI (*blue*). Outlined box in Merge (*white*) is enlarged in Detail panel. *Size bar* 2 μm. *n* = 3 experiments. **b** Polymerase chain reaction of mtDNA. MtDNA-specific primers were used to amplify nt7130-8113 of human mtDNA from 100 ng of total cellular DNA isolated from WT, ρ^0^, and CCCP-treated WT cells. *n* = 3 experiments. **c** Anti-MTCO2 Western blotting of lysates from cultured 143B cell lines. Anti-α-tubulin provides loading control. **d** Representative histograms of WT, ρ^0^, and CCCP-treated WT cells incubated with 100 nM TMRE and assayed via flow cytometry. 50,000 cells assayed for TMRE fluorescence (*X-axis*) in each experiment. *Y-axis* indicates number of cells at fluorescence values expressed in arbitrary units (a.u.). **e** Average TMRE fluorescence of WT, ρ^0^, and CCCP-treated WT cells in arbitrary units (a.u.). Values represent average TMRE fluorescence of indicated cell lines in *n* > 3 experiments ± standard error (SE). **Statistical significance, *p* < 0.01, Tukey’s post hoc test following one-way ANOVA

Previous studies have found that cells with OxPhos defects have complete fragmentation of the mitochondrial network [4, 16, 21]. To confirm these findings, we examined both genetic and pharmacological cell models of Δ*ψ*_m_ loss in a 143B osteosarcoma cell background. 143B206ρ^0^ cells have been depleted of all mtDNA through treatment with ethidium bromide (EtBr) [36], resulting in loss of mtDNA-encoded polypeptides, Δ*ψ*_m_, and OxPhos function [16, 19]. The protonophore CCCP allows for complete dissipation of the proton gradient across the mitochondrial inner membrane, providing a pharmacological method to eliminate Δ*ψ*_m_. To examine mitochondrial morphology in WT, ρ^0^, and CCCP-treated WT lines, cells were immunolabeled for the TOM20 protein, which is located in the mitochondrial outer membrane, providing imaging of the mitochondrial network in these cells. Mitochondrial localization was confirmed by colocalization with MitoTracker (not shown). WT cells visualized by confocal fluorescence microscopy revealed a balance of both fusion and fission, with highly interconnected, reticular mitochondria existing in the same cell with fragmented individual mitochondria. Conversely, ρ^0^ cells displayed fragmented, somewhat swollen, mitochondria, while WT cells treated with 10 μM CCCP for 1 h also showed complete fragmentation of the mitochondrial network (Fig. 1a). MtDNA content was verified by PCR from total DNA of indicated cell lines. Amplification of mtDNA nt7130-8113 revealed a strong band in both WT and CCCP-treated WT cells, while ρ^0^ cells showed no mtDNA present (Fig. 1b). Western blot analysis showed that the mtDNA-encoded cytochrome c oxidase II (MTCO2) protein was present in WT and CCCP-treated WT cells, but absent in ρ^0^ cells, as expected (Fig. 1c). Consistent with this, immunolabeling microscopy showed strong MTCO2 signal in WT and CCCP-treated WT cells, but not in ρ^0^ cells (not shown). To determine Δ*ψ*_m_, cells were given media containing the Δ*ψ*_m_-dependent dye tetramethyl rhodamine ester (TMRE) and assayed by flow cytometry (representative histograms, Fig. 1d). This assay is a novel adaptation of our previous method [19]. WT cells show a robust average fluorescence of 4172 ± 172 arbitrary units (a.u.), while ρ^0^s and CCCP-treated WT cells maintain significantly lower values of 1197 ± 179 a.u and 385 ± 26 a.u., respectively (Fig. 1e). These values are consistent with those reported for ρ^0^ cells via rhodamine-based evaluation of Δ*ψ*_m_ [21]. Collectively, these findings demonstrate that either genetic or pharmacological decreases in Δ*ψ*_m_ cause complete fragmentation of the mitochondrial network, consistent with previous findings [4, 16].

#### Cells below a threshold of 34% TMRE fluorescence cannot maintain mitochondrial interconnection

**Fig. 2:**
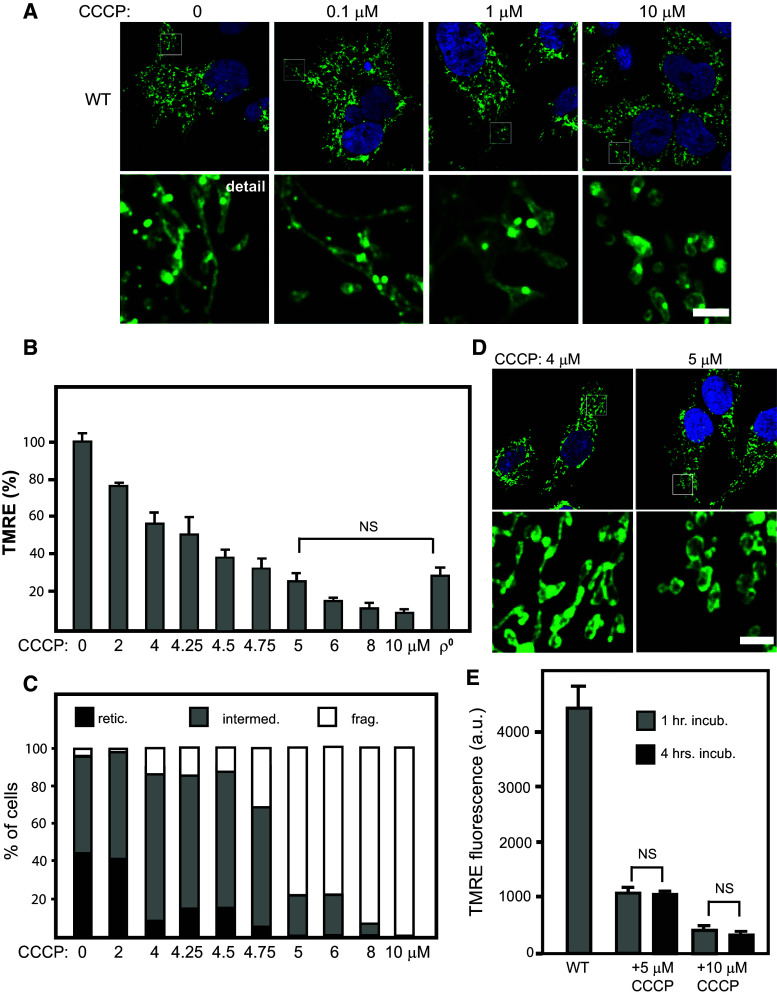
Mitochondrial morphology and Δ*ψ*_m_ monitored across CCCP titration. **a** WT cells incubated with 0, 0.1, 1, or 10 μM CCCP for 1 h were then fixed and immunolabeled for mitochondrial TOM20 (*green*), followed by DAPI staining of nuclei (*blue*). Outlined boxes (*white*) enlarged in Detail image. *Size bar* 2 μm. *n* = 3 experiments. **b** WT cells were incubated with 0, 2, 4, 4.25, 4.5, 4.75, 5, 6, 8, or 10 μM CCCP for 1 h, as well as ρ^0^ cells, and incubated with 100 nM TMRE for 20 min. Average TMRE fluorescence given for each condition, expressed as % of untreated (0 μM CCCP) ± SE. *n* ≥ 3 experiments for each condition, 50,000 cells assayed per experiment. All values analyzed by one-way ANOVA, *p* < 0.01. **c** Quantitation of mitochondrial morphology across CCCP titration. WT cells were incubated with CCCP as in **b** for 1 h and processed for TOM20 immunolabeling, as in **a**. In three independent experiments, cells were imaged and scored as having predominantly reticular (*black*), predominantly fragmented (*white*), or intermediate (*gray*) mitochondrial morphology. Average % of cells with indicated morphology shown across CCCP concentrations indicated. For analysis of statistical significance, see Table 1. **d** WT cells incubated in the presence of 4 or 5 μM CCCP and immunolabeled for TOM20 (*green*). *Size bar* 2 μm. **e** Cells were incubated in the absence or presence of 5 or 10 μM CCCP for 1 or 4 h and assayed by TMRE as in **b**. *n* ≥ 3 experiments for each condition, 50,000 cells assayed per experiment. All values analyzed by one-way ANOVA, *p* < 0.01. NS denotes *p* > 0.05, Tukey’s post hoc test

Intriguingly, ρ^0^ cells maintain a modest, intermediate TMRE signal, statistically greater than that of WT cells treated with 10 μM CCCP [1197 ± 179 versus 385 ± 26 a.u., respectively (Fig. 1e)], likely due to reversal of the F_1_F_0_ ATP synthase [37]. Despite this, both have completely fragmented mitochondrial morphology (Fig. 1a). This suggests that a minimum level of Δ*ψ*_m_, greater than that seen in either ρ^0^ or CCCP-treated WT cells, is required for reticular mitochondrial organization. To explore this, WT cells were incubated with a range of concentrations of CCCP for 1 h, and mitochondrial morphology visualized by anti-TOM20 immunolabeling, as above (Fig. 2a). At 0, 0.1, and 1 μM CCCP, cells displayed full ability to maintain mitochondrial interconnection, as reticular mitochondria were clearly evident. At 10 μM CCCP, however, no cells were observed with reticular mitochondria, as all cells had fragmented mitochondria (Fig. 2a), indicating that mitochondrial fission/fusion balance is lost between 1 and 10 μM CCCP. To further probe this hypothesis, cells were treated with CCCP from 2 to 10 μM, and TMRE flow cytometry was used to assay Δ*ψ*_m_ as in Fig. 1e. When normalized to untreated WT cells, CCCP at increasing concentrations produced stepwise decreases in TMRE fluorescence, defining a range from untreated (100%) to 10 μM CCCP-treated (9%). Critically, cells incubated with 4.75 μM CCCP showed a normalized TMRE signal of 34 ± 5% relative to untreated cells, while cells incubated with 5 μM CCCP had TMRE signal of 25 ± 5% and ρ^0^ cells 29 ± 4% (Fig. 2b). To explore the impact of these stepwise decreases in Δ*ψ*_m_ on mitochondrial morphology, cells treated with the indicated concentrations of CCCP were immunolabeled for TOM20. For quantitation, individual cells were scored as having predominantly reticular, fragmented, or intermediate mitochondrial morphologies, as elsewhere [34, 38–40]. When scoring cell profiles, individual cells with more than two regions of either reticular or fragmented mitochondrial organization were scored as intermediate. As expected, untreated cells showed a high proportion of cells with predominantly reticular (44 ± 17%) and intermediate morphologies (51 ± 15%), with a small (4 ± 3%) proportion of cells with predominantly fragmented mitochondria. On the other hand, cells treated with 10 μM CCCP showed 100% fragmented morphology (Fig. 2c; Table 1), demonstrating the spectrum of mitochondrial morphology and Δ*ψ*_m_ in 143B cells. Strikingly, cells with intermediate Δ*ψ*_m_ levels showed a clear threshold effect: at 0, 2, and 4 μM CCCP, >90% of cells assayed had either reticular or intermediate morphologies, with less than 15% of cells having a fragmented morphology at these concentrations. Conversely, at 5, 6, 8, and 10 μM, at least 80% of cells had predominantly fragmented mitochondrial morphology (Fig. 2c; Table 1). A sharp dropoff was observed between cells treated with 4.75 μM and 5 μM CCCP: at 4.75 μM CCCP, 70% of cells had either reticular or intermediate morphology, while cells treated with 5 μM CCCP showed 80% fragmented morphology (Fig. 2c; Table 1). Representative images demonstrate this striking threshold: at 4 μM CCCP, cells showed extensive regions of mitochondrial interconnection, while at 5 μM CCCP, the mitochondrial network was largely fragmented (Fig. 2d). Similar results were obtained for human HeLa and murine 3T3 cell lines: at 4 μM CCCP, both cell lines displayed clear evidence of mitochondrial interconnection when visualized by anti-TOM20 immunolabeling, while HeLa and 3T3 cells treated with 5 μM CCCP displayed near-complete fragmentation (Suppl. Figure 1). Strikingly, TMRE fluorescence of WT cells treated with 5 μM CCCP and untreated ρ^0^ cells are statistically equivalent [1023 ± 194 a.u. versus 1197 ± 197, respectively, not significant (NS) (Fig. 2b)], and both have fragmented mitochondrial morphology (Figs. 1, 2d). Additional experiments explored whether longer treatments with CCCP alter Δ*ψ*_m_ status: cells incubated with 5 μM CCCP for 1 versus 4 h showed equivalent TMRE values, as did cells incubated with 10 μM CCCP (Fig. 2e), indicating that increasing CCCP treatment beyond 1 h does not cause further decreases in Δ*ψ*_m_. This is consistent with findings elsewhere that indicate that protonophores such as CCCP act rapidly to dissipate Δ*ψ*_m_ [41], while longer incubations do not produce additional decreases in Δ*ψ*_m_ [42].

**Table 1:** Quantitation of mitochondrial morphology in CCCP-treated cells

CCCP (μM)	Reticular	Intermediate	Fragmented
0	44.4 ± 17	51.3 ± 15	4.3 ± 3
2	39.1 ± 10	58.9 ± 11	1.9 ± 1
4	5.7 ± 3*	83.5 ± 6	14.1 ± 7
4.25	15 ± 6	69 ± 4	16 ± 5
4.5	14.7 ± 2	72.3 ± 1	13 ± 3
4.75	4.7 ± 1	65 ± 3	30.3 ± 3
5	1 ± 1	19.6 ± 4**	79.6 ± 5**
6	0 ± 0	20.5 ± 8	79.5 ± 2
8	0 ± 0	5.6 ± 2	94.4 ± 2
10	0 ± 0	0 ± 0	100 ± 0

Values represent the average percentage of cells with the indicated morphology ± standard error in three independent experiments, >150 cells scored. Statistical analysis: data analyzed by one-way ANOVA, *p* < 0.05. * *p* < 0.05, ** *p* < 0.01, compared to value immediately above, Tukey’s post hoc test. These values were used to generate the bar graph of these data corresponding to Fig. 2c

As a Nernstian dye that is taken up by actively respiring mitochondria, TMRE has been extensively used to assay Δ*ψ*_m_ via flow cytometry and quantitative microscopy [43, 44]. Estimation of Δ*ψ*_m_ from TMRE or other fluorescence methods involves a range of factors, including the concentration of dye within the mitochondria, the cytosol, and the extracellular space, as well as the overall mass of mitochondria within the cell [44]. Moreover, protonophores such as CCCP impact the potential at both the mitochondria and the plasma membrane [45]. Gerencser et al. [46] found that the resting Δ*ψ*_m_ of cultured mammalian cells in high-glucose medium is −139 mV, while Springett et al. have successfully employed the oxidation state of mitochondrial bc1 heme groups to quantitatively explore Δ*ψ*_m_, but note the lack of additional direct methods to cross-correlate their measurements [47]. Given these caveats, discussion of Δ*ψ*_m_ levels are here restricted to TMRE fluorescence values, rather than extrapolating these differences to mV. TMRE values are normalized to untreated WT cells (Fig. 2c) to reflect the relative change between samples. Assuming this value as 100%, our results show that 4.75 μM CCCP, 5 μM CCCP, and ρ^0^ cells have relative TMRE levels of 34, 25 and 29%, respectively. These results indicate that both pharmacological and genetic loss of Δ*ψ*_m_ to levels below that of 4.75 μM-treated WT cells cause dramatic loss of reticular mitochondrial organization, representing a critical threshold required for mitochondrial fission/fusion balance. As mitochondrial dynamics are governed by distinct fission and fusion pathways, we next explored the involvement of OPA1, OMA1, and DRP1 in maintaining Δ*ψ*_m_-dependent mitochondrial dynamic balance.

### OMA1-mediated OPA1 cleavage is required for Δ*ψ*_m_-sensitive mitochondrial dynamic balance

**Fig. 3:**
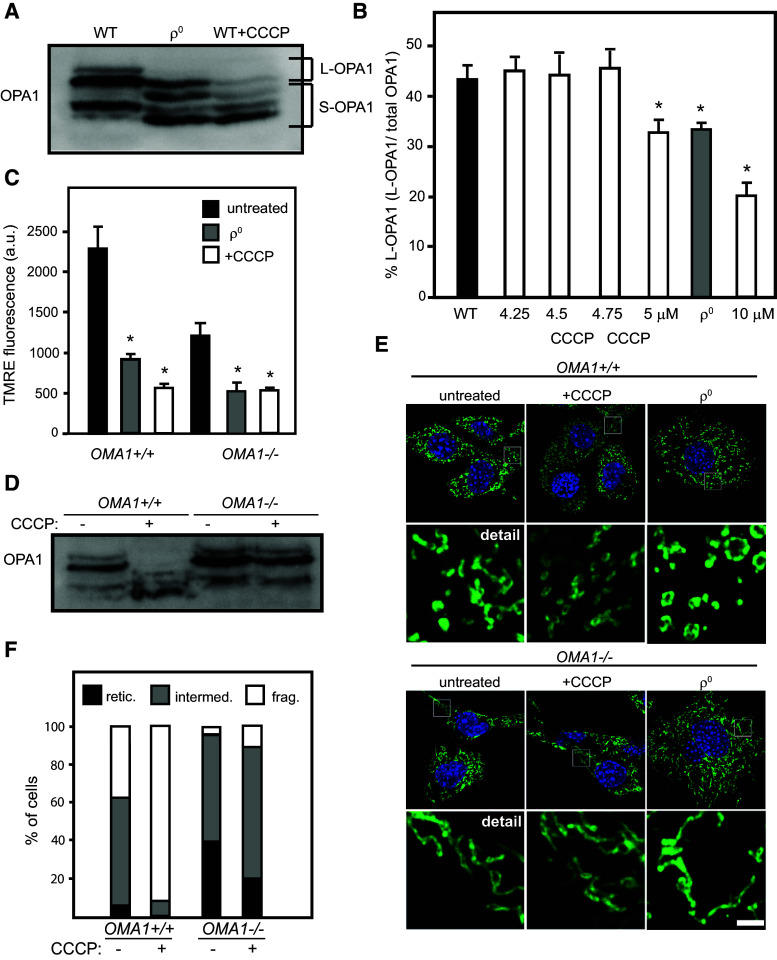
Role of OPA1 and OMA1 in Δ*ψ*_m_-dependent mitochondrial dynamics. **a** Anti-OPA1 Western blotting of 143B cells. Lysates were prepared from WT, ρ^0^, and 10 μM CCCP-treated WT cells, followed by blotting with anti-OPA1 monoclonal antibody. L-OPA1 (**a**, **b**) and S-OPA1 (**c**–**e**) isoforms labeled as indicated. *n* = 3 experiments. **b** Quantitation of Western blot densitometry using Image J, ± SE. *n* = 3 experiments. *Statistical significance, *p* < 0.05, one-way ANOVA followed by Dunnett’s post hoc test. **c** TMRE analysis of *OMA1* ^+*/*+^ and *OMA1* ^−*/*−^ mouse embryonic fibroblasts (MEFs). MEFs with (*OMA1* ^+*/*+^) and lacking (*OMA1* ^−*/*−^) the murine *OMA1* gene were incubated in the absence or presence of 20 μM CCCP for 1 h, or in the absence or presence of EtBr for 10 days. Untreated, CCCP-treated, or ρ^0^ MEFs were incubated with 100 nM TMRE and TMRE fluorescence quantitated via flow cytometry. Average TMRE fluorescence expressed in a.u. ± SE. *n* ≥ 3 experiments for each condition, 50,000 cells assayed per experiment. *Statistical significance, *p* < 0.01, one-way ANOVA followed by Dunnett’s post hoc test. **d** Anti-OPA1 Western blotting of lysates from *OMA1* ^+*/*+^ and ^−*/*−^ MEFs. MEFs were incubated in the absence or presence of 20 μM CCCP for 1 h and lysates were prepared, followed by blotting to PVDF and blotting with anti-OPA1. *n* = 3 experiments. **e** Mitochondrial morphology of *OMA1* ^+*/*+^ and *OMA1* ^−*/*−^ MEFs. Untreated, CCCP-treated (20 μM, 1 h), or mtDNA-depleted ρ^0^ MEFs in each background were immunolabeled for mitochondrial TOM20 (*green*) and counterstained with DAPI (*blue*). *n* = 3 experiments. *Size bar* 2 μm. **f** Quantitation of mitochondrial morphology *OMA1* ^+*/*+^ and *OMA1* ^−*/*−^ MEFs in the absence or presence of CCCP. As in Fig. 2c, cells were imaged and scored as having predominantly reticular (*black*), predominantly fragmented (*white*), or intermediate (*gray*) mitochondrial morphology. Average % of cells with indicated morphology in *n* = 3 experiments

As the mediator of mitochondrial inner membrane fusion, OPA1 has emerged as a key Δ*ψ*_m_-sensitive factor in mitochondrial dynamics. OPA1 exists as five isoforms in human cells (a–e): the a and b long isoforms (L-OPA1) mediate inner membrane fusion, while the c, d, and e short isoforms (S-OPA1) are fusion-inactive [48]. Dissipation of Δ*ψ*_m_ causes cleavage of fusion-mediating L-OPA1 isoforms to inactive S-OPA1 by the OMA1 protease, causing loss of mitochondrial fusion [32, 33], while expression of S-OPA1 activates mitochondrial fission [34]. We, therefore, next explored OPA1 status in ρ^0^ and CCCP-treated 143B models, as in Figs. 1 and 2. Anti-OPA1 Western blotting revealed that WT cells have prominent bands for the L-OPA1 a and b isoforms, with the fusion-inactive S-OPA1 showing a strong band for the d short isoform and minor bands for the c and e isoforms (Fig. 3a). ρ^0^ cells show dramatically decreased L-OPA1 isoforms, with increased levels of the c and e S-OPA1 isoforms. CCCP-treated WT cells show even more pronounced loss of L-OPA1 isoforms, with a near-complete loss of the a isoform and only a faint band for the b isoform, while the S-OPA1 isoforms are increased, with a major band for the e short isoform (Fig. 3a). Quantitation of OPA1 isoforms confirms this: untreated WT cells maintain 43 ± 3% L-OPA1 of total OPA1 signal, while ρ^0^ cells (33 ± 2% L-OPA1) and 10 μM CCCP-treated WT cells (20 ± 3%), show significant decreases in the proportion of L-OPA1 relative to untreated WTs. OPA1 blotting at intermediate CCCP concentrations mirrors the morphological threshold observed in Fig. 2c, d: WT cells treated with 4.25, 4.5, and 4.75 μM CCCP maintain similar L-OPA1 levels to untreated WT cells (45 ± 2, 44 ± 5, and 46 ± 4%, respectively), while WT cells treated with 5 μM CCCP have a significantly lower proportion of L-OPA1 (33 ± 3%) (Fig. 3b). These results suggest that >40% L-OPA1 (of total OPA1) is necessary for mitochondrial fusion capability, and further suggests that the Δ*ψ*_m_ threshold observed for mitochondrial interconnection (Fig. 2) reflects the available pool of fusion-active L-OPA1 isoforms.

As the OMA1 metalloprotease has emerged as a critical regulator of stress-sensitive OPA1-mediated mitochondrial fusion, we next examined the role of OMA1 in this process. While OPA1 is cleaved by a variety of proteases including YME1 [48–50], AFG3L1, and AFG3L2 [33], only OMA1 has been shown to mediate Δ*ψ*_m_-sensitive cleavage of OPA1 [32, 33], suggesting OMA1 as a likely regulator of the observed Δ*ψ*_m_ threshold of mitochondrial fission/fusion balance. To explore this, experiments examined pharmacological and genetic Δ*ψ*_m_ loss, as above, in mouse embryonic fibroblasts (MEFs) either carrying (*OMA1* ^+*/*+^) or ablated for (*OMA1* ^−*/*−^) the *OMA1* gene (kind gift of Dr. Carlos Lopez-Otin, University of Oviedo). When assayed via TMRE flow cytometry, *OMA* ^+*/*+^ cells show an average fluorescence of 2298 ± 294 a.u. Strikingly, *OMA1* ^−*/*−^ cells show a significantly decreased TMRE signal of 1227 ± 132 a.u., relative to *OMA1* ^+*/*+^ cells (Fig. 3c), indicating that these cells have a low basal Δ*ψ*_m_. Both *OMA1* ^+*/*+^ and *OMA1* ^−*/*−^ cell lines showed massive decreases in TMRE fluorescence when treated with 20 μM CCCP, with average fluorescence values of 567 ± 50 and 544 ± 21, respectively (Fig. 3c). Using EtBr treatment per King and Attardi [36], ρ^0^ MEF lines were generated in both *OMA1* ^+*/*+^ and *OMA1* ^−*/*−^ backgrounds. In both cases, the ρ^0^ line had a dramatically decreased Δ*ψ*_m_ relative to the control MEFs, as assayed by TMRE flow cytometry: *OMA1* ^+*/*+^ ρ^0^ cells had average TMRE fluorescence of 925 ± 42 versus 2298 ± 294 a.u. for control *OMA1* ^+*/*+^ cells, while *OMA1* ^−*/*−^ ρ^0^ cells had TMRE signal of 503 ± 118 a.u. versus 1227 ± 132 for control *OMA1* ^−*/*−^ cells (Fig. 3c). We next examined OPA1 status in *OMA1* ^+/+^ and *OMA1* ^−*/*−^ cells in the absence or presence of CCCP. While untreated *OMA1* ^+*/*+^ and *OMA1* ^−*/*−^ cells showed equivalent levels of L- and S-OPA1 isoforms, CCCP-treated *OMA1* ^+*/*+^ cell lysates showed loss of L-OPA1 isoforms and concomitant increase in S-OPA1 isoforms. Conversely, *OMA1* ^−*/*−^ cells treated with CCCP showed no change in OPA1 isoforms versus untreated *OMA1* ^−*/*−^ control cell lysates (Fig. 3d).

When examined by anti-TOM20 immunolabeling, *OMA1* ^+*/*+^ MEFs show the expected mix of fragmented and reticular morphologies (Fig. 3d). Conversely, *OMA1* ^−*/*−^ cells show extensive interconnection (Fig. 3d), indicating that cells lacking the OMA1 metalloprotease retain full mitochondrial fusion capability, despite a low basal Δ*ψ*_m_. When treated with CCCP, *OMA1* ^+*/*+^ cells show the expected fragmented morphology, while *OMA1* ^−*/*−^ cells retain extensive mitochondrial interconnection in the presence of CCCP (Fig. 3d), consistent with findings elsewhere [51, 52]. Similarly, *OMA1* ^+*/*+^ ρ^0^ cells show a fragmented morphology, consistent with the results in Figs. 1 and 2, and previous findings [16, 18]. Strikingly, however, *OMA1* ^−*/*−^ ρ^0^ cells show extensive mitochondrial interconnection (Fig. 3d), despite the loss of Δ*ψ*_m_. Quantitation of morphology confirms this: while *OMA1* ^+*/*+^ cells show dramatic loss of reticular and intermediate morphologies when treated with CCCP, compared with untreated *OMA* ^+*/*+^ cells, *OMA1* ^−*/*−^ cells do not show a significant change in the proportion of cells with reticular or intermediate morphologies when treated with CCCP (Fig. 3f). These results demonstrate that cells lacking OMA1 are insensitive to loss of Δ*ψ*_m_, retaining a fused mitochondrial network when challenged with either pharmacological or genetic loss of Δ*ψ*_m_. However, we note that CCCP-treated *OMA1* ^−*/*−^ cells show an overall decrease in the proportion of cells’ reticular mitochondria (21 ± 13 versus 40 ± 3% for untreated *OMA1* ^−*/*−^ cells) (Fig. 3f). While this decrease is not statistically significant, it does suggest that additional factors may be involved.

**Fig. 4:**
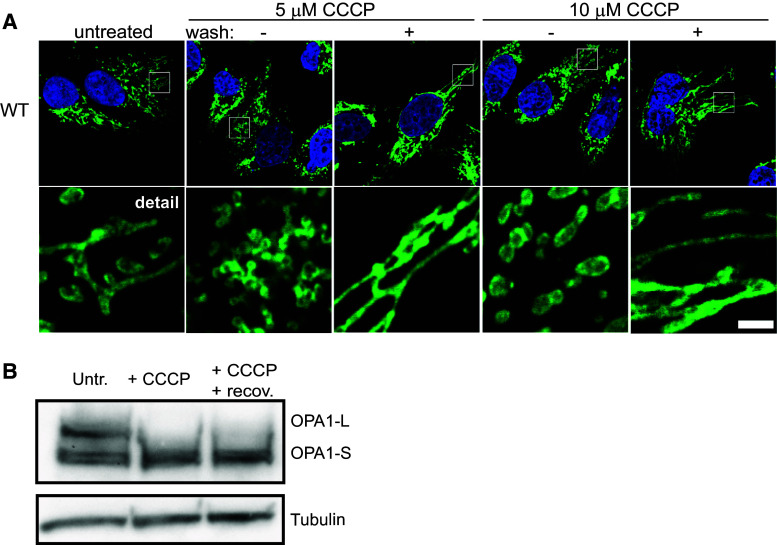
CCCP treatment and recovery restores mitochondrial interconnection. **a** WT cells were incubated with either 5 or 10 μM CCCP for 1 h without or with subsequent recovery in media lacking CCCP (wash) for 3.5 h [48] followed by anti-TOM20 immunolabeling, *n* = 3 experiments. **b** Western blotting of WT cells treated as in **a**. Lysates were prepared from untreated WT, 10 μM CCCP-treated WT cells, and 10 μM CCCP-treated WT cells allowed to recover in CCCP-free media, followed by blotting with anti-OPA1 and anti-tubulin monoclonal antibodies. L-OPA1 (**a**, **b**) and S-OPA1 (**c**–**e**) isoforms labeled as indicated. *n* = 3 experiments

These findings strongly indicate that L-OPA1 is a key determinant of the Δ*ψ*_m_-dependent mitochondrial fission/fusion dynamics observed in Fig. 2. To explore this further, we incubated WT cells with 5 and 10 μM CCCP, as in Fig. 2, followed by recovery in CCCP-free media, and examined mitochondrial morphology. Untreated WT cells maintained the expected balance of mitochondrial fission and fusion, while cells incubated with 5 and 10 μM CCCP both showed the extensive mitochondrial fragmentation noted in Fig. 2 (Fig. 4a). When CCCP-treated cells (either 5- or 10 μM-treated) were allowed to recover in fresh media lacking CCCP, extensive mitochondrial interconnection was observed in both, with highly elongated, networked mitochondria observed (Fig. 4a). Western blotting of CCCP-treated cells showed the expected loss of L-OPA1 isoforms, while CCCP-treated cells allowed to recover in CCCP-free media failed to restore L-OPA1 (Fig. 4b), consistent with the results of Griparic et al. [48]. Thus, while the results of Fig. 3 indicate that L-OPA1 levels correlate with the observed fusion threshold, with OMA1 playing a major role in mediating Δ*ψ*_m_-dependent mitochondrial dynamic balance, Fig. 4 shows that extensive mitochondrial interconnection can nevertheless be demonstrated in the absence of an abundant pool of fusion-active L-OPA1. Similarly, CCCP-treated *OMA1* ^−*/*−^ cells show a decreased (though not statistically significant) percentage of cells with reticular morphology, relative to untreated *OMA1* ^−*/*−^ cells (Fig. 3f). Taken together, these findings suggest that other factors likely play a role in Δ*ψ*_m_-dependent mitochondrial dynamic balance. Notably, DRP1 is actively recruited to mitochondria of CCCP-treated cells [53], suggesting that CCCP treatment and recovery cause effective inhibition of DRP1-mediated fission, causing the observed extensive mitochondrial interconnection in Fig. 4. We, therefore, next explored the contribution of DRP1 to Δ*ψ*_m_-dependent mitochondrial dynamic balance.

### DRP1 is required for Δ*ψ*_m_-dependent mitochondrial dynamic balance

**Fig. 5:**
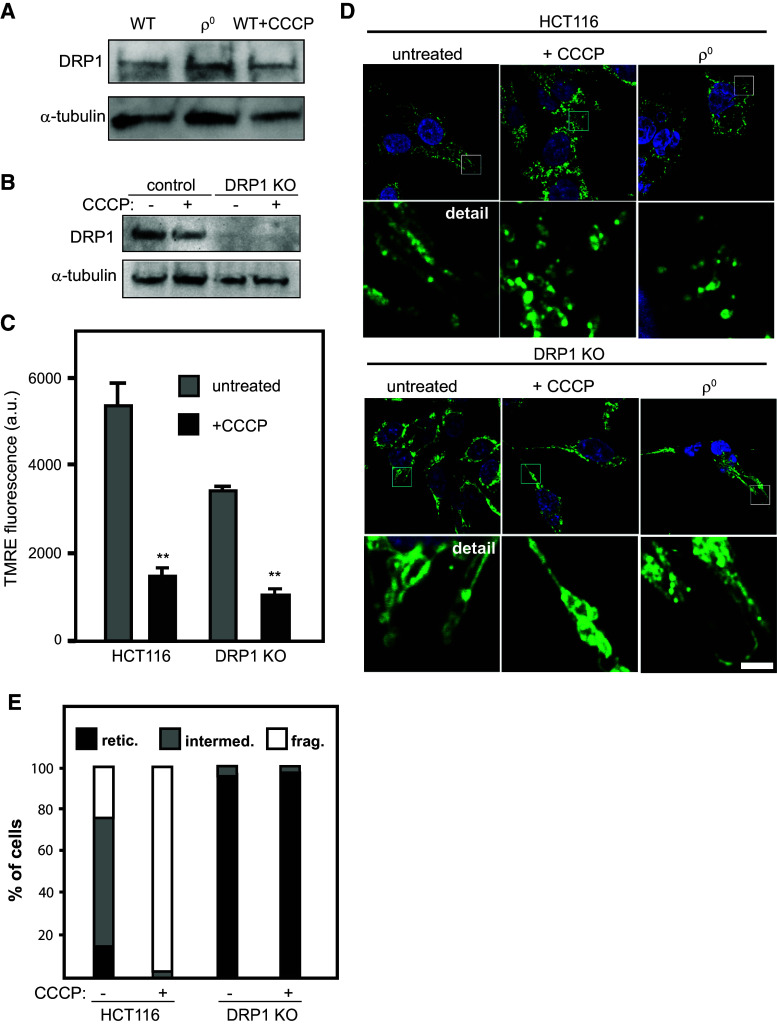
Δ*ψ*_m_-sensitive mitochondrial dynamics require DRP1. **a** Anti-DRP1 Western blotting of lysates from cultured 143B cell lines. Anti-α-tubulin provides loading control. *n* = 3 experiments. **b** Anti-DRP Western blotting of control and DRP1 knockout HCT116 cells without or with CCCP treatment (10 μM, 1 h). Anti-α-tubulin provides loading control. *n* = 3 experiments. **c** TMRE flow cytometry of HCT116 and DRP1 knockout cell lines in the absence or presence of CCCP (10 μM, 1 h). Average TMRE fluorescence expressed in a.u. ± SE. *n* ≥ 3 experiments for each condition, 50,000 cells assayed per experiment. **Statistical significance from corresponding untreated cell lines, *p* < 0.01, Tukey’s post hoc test following one-way ANOVA. **d** Immunofluorescence of HCT116 control and DRP1 knockout cells. Untreated, CCCP-treated (10 μM, 1 h), and mtDNA-depleted ρ^0^ cells in each background were immunolabeled for TOM20 (*green*) and stained with DAPI (*blue*). Outlined boxes (*white*) enlarged in Detail image. *Size bar* 2 μm. *n* = 3 experiments. **e** Quantitation of mitochondrial morphology in HCT116 and DRP1 knockout cells in the absence or presence of CCCP. Cells were imaged and scored as having predominantly reticular (*black*), predominantly fragmented (*white*), or intermediate (*gray*) mitochondrial morphology, as above (Figs. 2c, 3f). Average % of cells with indicated morphology in *n* = 3 experiments

To further explore the mechanism of Δ*ψ*_m_-dependent mitochondrial dynamics, we next tested the role of DRP1-mediated fission in this process. Western blotting for DRP1 in WT, ρ^0^, and CCCP-treated WT cell lysates revealed no appreciable differences in DRP1 expression between the different cell lines (Fig. 5a). To explore the functional role of DRP1 in Δ*ψ*_m_-sensitive fission/fusion balance, we examined Δ*ψ*_m_ loss under both pharmacological inhibition and genetic knockout of DRP1. Mitochondrial morphology was examined in WT and ρ^0^ cells in the presence or absence of mdivi-1, a small molecular agent that prevents the oligomerization of DRP1 at the mitochondrial outer membrane [13]. Untreated WT cells displayed the expected mixture of fusion and fission, while many mdivi-1-treated WT cells showed elaborately interconnected mitochondria, as visualized using MitoTracker (Suppl. Figure 2A). Quantitation of morphology, as in Fig. 2, reflected this: 36 ± 3% of untreated WT cells had a predominantly reticular morphology, but mdivi-1 treatment significantly increased this to 56 ± 6%. Similarly, the proportion of cells with fragmented mitochondria significantly decreased, falling from 11 ± 1% in untreated WT cells to 4 ± 2% in response to mdivi-1 (Suppl. Figure 2B). These results demonstrate that mdivi-1 inhibits fission in this system, causing increased mitochondrial fusion. Untreated ρ^0^ cells show a completely fragmented mitochondrial ultrastructure, as in Fig. 1. Strikingly, however, mdivi-1-treated ρ^0^ cells also show a completely fragmented morphology, confirmed by quantitation: no ρ^0^ cells were observed to have a reticular morphology in either the absence or presence of mdivi-1, (Suppl. Figure 2A, B). Similar results were obtained in CCCP-treated WT cells: mdivi-1-treated WT cells challenged with 5 or 10 μM CCCP also showed total fragmentation of the mitochondrial network, with no observable organellar interconnection (Suppl. Figure 2C), suggesting that Δ*ψ*_m_-dependent fragmentation of the mitochondrial network might be independent of DRP1-mediated fission.

However, mdivi-1 may not be sufficiently potent to completely inhibit DRP1 fission activity, particularly if loss of Δ*ψ*_m_ stimulates DRP1 functional activity, as found elsewhere [39] via phosphorylation at residue Ser656 [54]. To more rigorously test the requirement for DRP1 in Δ*ψ*_m_-sensitive mitochondrial dynamics, we examined human HCT116 cells containing or knocked out for DRP1 (kind gift of Dr. Richard Youle, NIH) with pharmacological (CCCP) or genetic (mtDNA-depleted) loss of Δ*ψ*_m_. Anti-DRP1 Western blotting of HCT116 control and DRP1 knockout cells confirmed that DRP1 is present in both untreated and CCCP-treated HCT116 cells, while DRP1 knockout cells (without or with CCCP treatment) lack DRP1 (Fig. 5b). TMRE flow cytometry was used as above to monitor Δ*ψ*_m_: HCT116 cells maintained a TMRE signal of 5368 ± 551 a.u. while CCCP treatment lowered their TMRE value to 1438 ± 230 a.u. DRP1 knockout cells, however, had an average TMRE fluorescence of 3379 ± 85 a.u., significantly lower than control HCT116s, consistent with bioenergetic functional defects in cells with genetically altered fission or fusion dynamics [55]. CCCP-treated DRP1 knockout cells showed a dramatic decrease in TMRE signal to 1092 ± 72 a.u., confirming that CCCP dissipates Δ*ψ*_m_ in HCT116 and DRP1KO cells (Fig. 5c). Mitochondrial morphology of HCT116 and DRP1KO cells was monitored via confocal microscopy with anti-TOM20 immunolabeling, as above. While control HCT116 cells maintained a largely intermediate morphology, with regions of both mitochondrial interconnection and fragmentation, DRP1 knockout cells showed elaborately interconnected mitochondria, as visualized by TOM20 imaging (Fig. 5d). In response to either CCCP treatment or depletion of mtDNA (via EtBr treatment), control HCT116 cells show fragmentation of the mitochondrial network, as in Fig. 1. Conversely, DRP1 knockout cells maintain an extensively interconnected mitochondrial network when treated with CCCP or under mtDNA depletion (Fig. 5d). Quantitation of morphology, as in Figs. 2c and 3f, supports this: CCCP-treated HCT116 cells show a dramatic increase in cells with fragmented mitochondria (98 ± 1%) compared with untreated HCT116s (24 ± 6%). Conversely, DRP1 knockout cells show overwhelmingly reticular morphology (96 ± 2%) that does not change in response to CCCP treatment (97 ± 2%). These results demonstrate that DRP1 knockout cells are insensitive to loss of Δ*ψ*_m_, and indicate that DRP1 plays a major role in maintaining Δ*ψ*_m_-dependent mitochondrial fission/fusion balance.

### DRP1 and OMA1 coordinately impact mitochondrial fusion and OPA1

Collectively, our findings indicate that both OMA1 and DRP1 are required for Δ*ψ*_m_-dependent mitochondrial dynamic balance, as cells lacking either factor maintain mitochondrial interconnection when challenged with loss of Δ*ψ*_m_ (Figs. 3, 5). Moreover, WT cells challenged with CCCP followed by recovery in CCCP-free media show extensive reticular mitochondria, despite the lack of abundant L-OPA1 in these cells (Fig. 4). These findings suggest that OMA1 and DRP1 cooperatively act to mediate Δ*ψ*_m_-dependent mitochondrial fusion/fission balance.

**Fig. 6:**
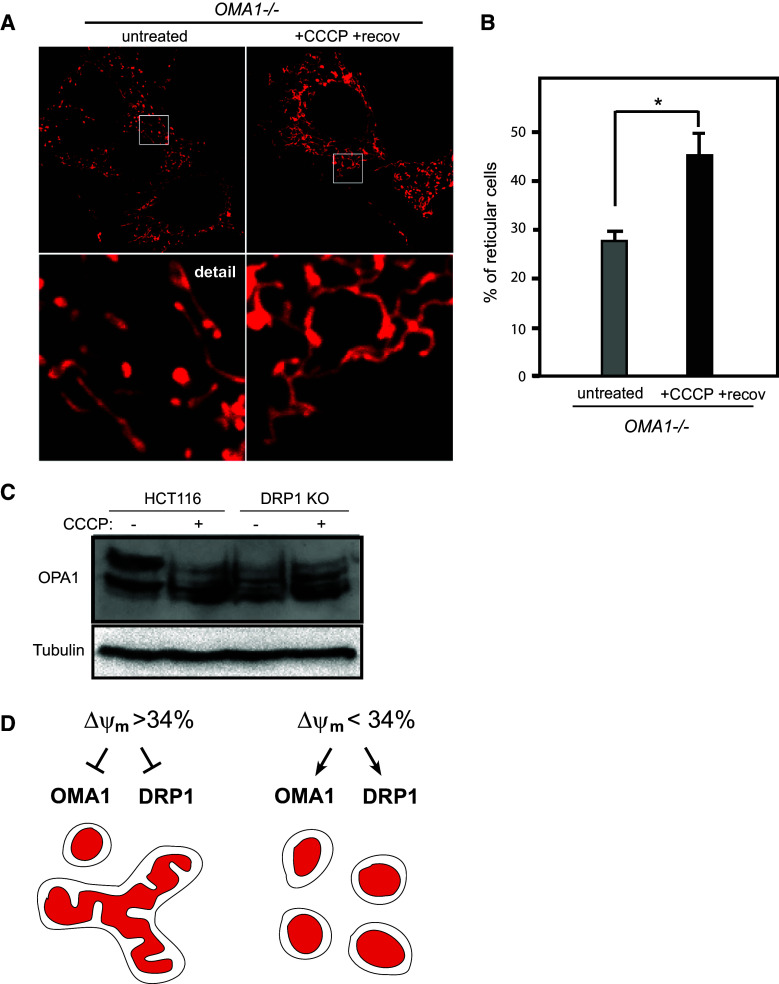
Cooperative roles for OMA1 and DRP1 in Δ*ψ*_m_-dependent mitochondrial dynamics. **a** Untreated and CCCP-treated + recovery *OMA1* ^−*/*−^ cells were incubated with MitoTracker and visualized by confocal microscopy, *n* = 3 experiments. **b** Quantitation of mitochondrial morphology of cells in **a**. Percentage of cells with predominantly reticular morphology in untreated versus CCCP-treated + recovery *OMA1* ^−*/*−^ cells, *n* = 3 experiments. *Statistical significance, *p* < 0.01, Student’s *t* test. **c** Anti-OPA1 blotting of control and DRP1 knockout HCT116 cells. Cells were incubated in the absence or presence of 10 μM CCCP for 1 h and lysates were prepared, followed by blotting with anti-OPA1. *n* = 3 experiments. **d** Model of Δ*ψ*_m_-dependent fission/fusion balance. An intact Δ*ψ*_m_ (at or above the 34% TMRE threshold) acts to inhibit both L-OPA1 cleavage by OMA1 and recruitment of DRP1 to mitochondria, resulting in intact mitochondrial fusion. When Δ*ψ*_m_ falls below the observed threshold, fission is activated via increased DRP recruitment to the mitochondria, while OMA1 proteolytic activity is activated, rapidly depleting L-OPA1, resulting in a completely fragmented population of individual organelles

To explore this possibility, we hypothesized that CCCP challenge and recovery would cause an additive increase in mitochondrial interconnection even in the presence of a stable pool of L-OPA1. To test this, we challenged *OMA1* ^−*/*−^ cells with CCCP treatment and recovery (in CCCP-free media) as in Fig. 4. Untreated *OMA1* ^−*/*−^ cells showed the expected mixture of reticular and intermediate morphologies expected, as per Fig. 3. Strikingly, however, *OMA1* ^−*/*−^ cells challenged with CCCP and recovery showed a robust increase in reticular mitochondrial morphology, with elaborately interconnected mitochondria apparent (Fig. 6a). Quantitation confirmed this: while 28 ± 2% of untreated *OMA1* ^−*/*−^ cells had a reticular mitochondrial morphology, OMA1^−*/*−^ cells given CCCP challenge and recovery showed a significant increase, with 45 ± 5% of cells showing a reticular mitochondrial morphology. Thus, despite the presence of a stable pool of available Δ*ψ*_m_-independent L-OPA1 (Fig. 3d), *OMA1* ^−*/*−^ cells show an additive increase in mitochondrial interconnection when challenged with CCCP and recovery (Fig. 6a, b), strongly indicating that DRP1 and OMA1 together modulate mitochondrial dynamic balance. While OMA1 has been extensively characterized as a key mediator of Δ*ψ*_m_-dependent OPA1 cleavage [32–34], we examined OPA1 status in DRP1 knockout and control cells to see whether DRP1 impacts OPA1.

To explore this, we examined HCT116 control and DRP1 knockout cells via OPA1 Western blotting. Control HCT116 cells show the expected distribution of OPA1 isoforms, with prominent bands for the b and d isoforms. Upon incubation with CCCP, HCT116 cells show cleavage of L-OPA1 isoforms, resulting in accumulation of S-OPA1 isoforms (Fig. 6c), consistent with the mitochondrial fragmentation found in CCCP-treated HCT116s in Fig. 5d. Untreated DRP1 knockout cells show the five OPA1 isoforms, with comparatively lower L-OPA1 levels than control HCT116 cells. Strikingly, however, DRP1 knockout cells treated with CCCP show no appreciable change in OPA1 status: L- and S-OPA1 levels are essentially identical in control versus CCCP-treated DRP1 knockout cells. This is consistent with the extensive mitochondrial interconnection shown in CCCP-treated DRP1 knockouts (Fig. 5d, e). While DRP1 knockouts have decreased L-OPA1 relative to control HCT116s, they do not show any activation of OPA1 cleavage in response to CCCP treatment (Fig. 6c). These findings are consistent with loss of L-OPA1 in response to DRP1 downregulation [56], and suggest that the DRP1-mediated fission machinery interacts with OPA1 and the mitochondrial fusion factors to cooperatively mediate Δ*ψ*_m_-dependent mitochondrial dynamic balance.

## Discussion

The interaction of bioenergetic function with fusion and fission pathways drives the structure/function dynamics of mitochondria as an organellar network. While it has been shown that complete dissipation of Δ*ψ*_m_ causes mitochondrial fragmentation, we here examine (1) the level of Δ*ψ*_m_ required, and (2) the contribution of both fission and fusion pathways, in maintaining Δ*ψ*_m_-dependent mitochondrial dynamic balance in human cells. Our findings indicate that a threshold of 34% TMRE-assayed Δ*ψ*_m_, strongly correlating with available fusion-active L-OPA1, is required to maintain mitochondrial fission/fusion balance. Surprisingly, cells lacking either OMA1 or DRP1 have obligate mitochondrial fusion and fail to cleave long OPA1 isoforms in response to pharmacological or genetic loss of Δ*ψ*_m_. These results suggest that OMA1 and DRP1 cooperatively act to mediate mitochondrial Δ*ψ*_m_-dependent dynamic balance, and provide further evidence in support of a proposed mechanistic link between DRP1-mediated fission and OPA1 [34, 57]. As mitochondrial dynamic balance is crucial to cellular processes such as apoptosis, this ‘tipping point’ mechanism of mitochondrial dynamic balance will have far-reaching impacts on these key pathways.

### Mitochondrial dynamic balance requires a threshold of Δ*ψ*_m_

Here, we address a key question of mitochondrial structure/function relationships: to what extent can the mitochondrial network lose bioenergetic function before dynamic balance is compromised? While total collapse of Δ*ψ*_m_ has been shown to cause fragmentation of the mitochondrial network [4, 32, 33], the functional tipping point of Δ*ψ*_m_-dependent mitochondrial dynamics has remained unknown. Mitochondrial threshold effects have been extensively documented in mtDNA-derived neuromuscular diseases: cells or tissues carrying greater than 80–90% mutant mtDNA typically show collapse of bioenergetic capacity [30]. Previously, we found that cells carrying a mutation load greater than 90% ∆-mtDNA lost the ability to maintain mitochondrial interconnection [17]. Here, we explore the functional and mechanistic tipping point behind this threshold using TMRE flow cytometry to assay Δ*ψ*_m_, and confocal imaging to monitor mitochondrial organization. When normalized against the TMRE value of untreated WT cells as 100%, our data indicate that cells below 34% TMRE signal have disrupted fission/fusion homeostasis. CCCP titration demonstrates that at concentrations above 4.75 μM, cells have obligate fragmentation of the mitochondrial network (Fig. 2). This loss of mitochondrial interconnection correlates with loss of fusion-active L-OPA1 isoforms (Fig. 3a, b), strongly indicating that OPA1-mediated inner membrane fusion per se is lost beyond this point. These findings demonstrate a key bioenergetic determinant of mitochondrial fission/fusion dynamics. Further research may employ alternate methods such as live-cell imaging [43] and heme oxidation state [47] to confirm the Δ*ψ*_m_ threshold in mV. In addition, this Δ*ψ*_m_ threshold likely contributes to the canonical mitochondrial genetic threshold effect: our model predicts that any mtDNA mutation that pushes Δ*ψ*_m_ below this threshold commits the organelle to obligate fission. As such, this parameter is likely to be of critical importance to mitochondrial pathology in a range of human diseases, as well as therapeutic strategies seeking to eliminate Δ*ψ*_m_-impacting mutant mtDNAs through pathways such as autophagy [58].

### OMA1 and DRP1 coordinately mediate Δ*ψ*_m_-dependent mitochondrial dynamics

As mitochondrial fission and fusion pathways have distinct sets of mediating factors, either pathway (or both) could be mechanistically responsible for this tipping point threshold. Mitochondrial fusion has been shown to be dependent on Δ*ψ*_m_ [4], mediated by L-OPA1 isoforms [48] that are cleaved to fusion-inactive S-OPA1 by OMA1 in response to loss of Δ*ψ*_m_ [32, 33]. These findings suggested that fusion is the major mediator of Δ*ψ*_m_-dependent mitochondrial dynamics, somewhat de-emphasizing the role of DRP1-mediated fission. However, our results show that cells lacking either OMA1 or DRP1 are insensitive to loss of Δ*ψ*_m_. If OMA1 alone controlled Δ*ψ*_m_-dependent mitochondrial dynamics, DRP1 knockout cells would fragment in ρ^0^ or CCCP-treated lines. The highly interconnected mitochondrial morphology of DRP1 knockout cells (Fig. 4) despite their lack of abundant L-OPA1 (Fig. 5) demonstrates the importance of DRP1. Similarly, cells treated with CCCP, with subsequent recovery in fresh media, do not show restoration of L-OPA1 (Fig. 4b), but nevertheless show striking mitochondrial interconnection (Fig. 4a). Taken together, these data support a strong role for DRP1 and fission per se in maintaining Δ*ψ*_m_-dependent mitochondrial dynamic balance.

Moreover, our results support both functional coordination and mechanistic interaction of OPA1-mediated fusion and DRP1-mediated fission. While experiments in cells lacking OMA1 (Fig. 3) or DRP1 (Fig. 5) show that both are necessary for mitochondrial dynamic balance, the decreased L-OPA1 observed in DRP1 knockout cells (Fig. 6c) indicates that DRP1 is necessary to maintain L-OPA1 stability. This enhanced OPA1 processing is consistent with Mopert et al. [57], who found similar results in response to transient knockdown of DRP1, while Huang et al. found that DRP interacts with mitofusin 2 [59], consistent with DRP1 interactions with the mitochondrial fusion machinery. These findings suggest a role for DRP1 in stabilizing mitochondrial fission/fusion factors via direct and indirect protein–protein interactions spanning the outer and inner mitochondrial membranes.

Thus, while mitochondrial fission and fusion have frequently been discussed as mechanistically distinct pathways, these results support a model in which an intact (i.e., above threshold) Δ*ψ*_m_ provides a check to both OMA1 and DRP1, allowing a balance of fission and fusion with both DRP1 and OMA1 interacting (directly or indirectly) with OPA1. Loss of Δ*ψ*_m_ to levels below the observed 34% TMRE threshold simultaneously activates OMA1 cleavage of L-OPA1 and mitochondrial recruitment of DRP1, collapsing mitochondrial organization to an obligately fragmented state (Fig. 6d). It is likely that the loss of Δ*ψ*_m_ below threshold causes profound changes in conformation and enzymatic activity of multiple proteins at the mitochondrial inner and outer membranes, altering the protein–protein interactions that determine mitochondrial structural dynamics. OMA1 has been shown to undergo self-cleavage and activation of L-OPA1 proteolysis during membrane depolarization [60], while Anand et al. found that expression of S-OPA1 is sufficient to induce mitochondrial fission, with S-OPA1 frequently colocalizing with DRP1 at ER-mitochondrial sites of contact [34]; the authors postulated that S-OPA1 may stimulate DRP1 fission activity. Δ*ψ*_m_-dependent DRP1 fission activity is activated by phosphorylation at S637 [27], and can be activated by calcineurin in response to increased cytosolic calcium [53]. Recruitment of DRP1 to mitochondria is mediated by actin [61, 62]. Taken together, these findings indicate that Δ*ψ*_m_-dependent mitochondrial structural homeostasis involves the highly dynamic realignment and recruitment of multiple factors in different mitochondrial and cellular compartments.

Mitochondrial fission/fusion status directly impacts a broad range of cellular processes such as apoptosis. Consistent with this, cells below the Δ*ψ*_m_ threshold show dramatically decreased viability when challenged with galactose-containing media (Suppl. Figure 3). As such, this tipping point threshold of Δ*ψ*_m_-dependent fission/fusion balance may have major impacts on cell-wide signaling events including autophagy, mitosis, and nutrient utilization. For example, collapse of Δ*ψ*_m_ is a key step in mitochondrial autophagy [63], causing recruitment of the Parkin E3 ubiquitin ligase to the mitochondria and targeting the organelle to the autophagosome [64], while mitochondrial fission is directly activated by AMP kinase signaling [65]. These findings thus have direct relevance to a wide range of prevalent diseases that include mitochondrial dysfunction in their pathogenesis. Mitochondrial dynamics are increasingly found to be critical for energetically dependent tissues such as heart [66] and skeletal muscle [67], with decreased OxPhos function and disrupted fission/fusion balance emerging in patient samples and disease models of neuromuscular diseases [30], neurodegenerative aging and Parkinson’s disease [68, 69], diabetes [70–72], and heart failure [28]. As such, our findings illustrate a basic mechanism of mitochondrial structure/function homeostasis with major implications for the pathogenesis and translational treatment of these disorders.

## Materials and methods

### Cell culture

Human 143B osteosarcoma cell lines FLP6a39.2 (WT) and 143B206 (ρ^0^) were described previously [16, 19], as have *OMA1* ^+*/*+^ and *OMA1* ^−*/*−^ mouse embryonic fibroblasts (MEFs) [52] and human colorectal carcinoma HCT116 control and DRP1 knockout cell lines [73]. Cells were grown in high-glucose Dulbecco’s Modified Eagle’s medium (DMEM) with 10% fetal bovine serum supplemented with 50 μg/mL uridine in 5% CO_2_ at 37 °C. CCCP and mdivi-1 (Sigma, St. Louis, MO, USA) were dissolved as stock solutions in DMSO for dilution in complete media. All cell culture reagents were from ThermoFisher (Waltham, MA, USA).

### Fluorescence microscopy

Cultured cells were seeded to 18 mm^2^ glass coverslips. Coverslips were incubated with MitoTracker CMXRos (Invitrogen Molecular Probes, Carlsbad, CA, USA), followed by fixation in 4% paraformaldehyde in PBS. For immunolabeling of the translocase of the outer mitochondrial membrane-20 protein (TOM20), cells were permeabilized with 0.1% TX-100 in PBS, followed by blocking in 10% normal goat serum and incubation with anti-TOM20 monoclonal antibody FL-145 at 1:100 dilution (Santa Cruz Biotechnology, Santa Cruz, CA, USA). Coverslips were incubated with goat anti-mouse Alexa488-conjugated secondary antibody at 1:100 dilution (Invitrogen Molecular Probes, Carlsbad, CA, USA), followed by staining with diaminophenylindole (DAPI) and mounting in 50% glycerol in PBS. Coverslips were imaged on an Olympus Fluoview FV-10i (Olympus, Center Valley, PA, USA) with a 60× UPLSAP60xW objective with aperture 1.2 and 3× optical zoom at room temperature. For scoring of mitochondrial morphology, individual cell profiles on confocal micrographs were scored as predominantly reticular if they had fewer than three instances of fragmented mitochondria. Cells were scored as predominantly fragmented if they displayed fewer than three instances of mitochondrial interconnection. All others were scored as having intermediate mitochondrial morphology.

### Flow cytometry

To assay Δ*ψ*_m_ using flow cytometry, we modified our previous method using the Δ*ψ*_m_-specific dye tetramethyl rhodamine ester (TMRE) [19]. Briefly, cells were seeded in 100-mm dishes and incubated with 100 nM TMRE for 20 min, followed by trypsinization and two washes in PBS. Cells were resuspended in 1 mL of PBS and analyzed using a BD Biosciences LSR Fortessa.

### PCR analysis of mtDNA

Total cellular DNA was isolated using proteinase K digestion, followed by phenol/chloroform extraction and ethanol precipitation, as previously [16]. Forward primer ACGCCAAAATCCATTTCACT and reverse primer CGGGAATTGCATCTGTTTTT amplify nt7130-8113 of human mtDNA. Reactions were electrophoresed on a 1% agarose gel and imaged using a Fotodyne Foto/Eclipse gel documentation system.

### Western blot analysis

Cells were lysed using a modified RIPA buffer on ice, and lysates were run on a 10% SDS-PAGE gel, followed by transfer to Immobilon PVDF membrane (Bio-Rad, Redmond, WA, USA). Anti-OPA1 blotting used separation of proteins on a 6% SDS-PAGE gel, per Griparic et al. [48]. Membranes were blocked in 5% milk in TBS overnight at 4 degrees C, followed by incubation with anti-MTCO2 monoclonal antibody ab110258 at 1:1000 dilution (Abcam, Cambridge, MA, USA), anti-DRP1 monoclonal ab56788 at 1:1000 dilution (Abcam, Cambridge, MA, USA), anti-OPA1 monoclonal antibody 612606 at 1:500 dilution (BD Biosciences, San Jose, CA, USA) and goat anti-mouse poly-HRP secondary antibody at 1:3000 dilution. Blots were developed using WestDura chemiluminescent reagents and imaged using a Kodak 4000MM Image Station or Bio-Rad ChemiDoc XRS. Anti-α-tubulin monoclonal antibody (Sigma) was employed as a loading control.

### Electronic supplementary material

Below is the link to the electronic supplementary material.

Supplemental Fig. 1. Δ*ψ*_m_ threshold of mitochondrial interconnection is a general phenomenon, and is reversible. Human HeLa and murine 3T3 cell lines were incubated in the absence or presence of CCCP at the indicated concentrations and visualized by anti-TOM20 immunolabeling. n = 3 experiments. Size bar = 2 μm (PDF 5764 kb)

Supplemental Fig. 2. Mdivi-1 does not increase fusion in cells with low Δ*ψ*_m_. A. WT and ρ^0^ cells were stained with MitoTracker (red) without (-) or with (+) overnight pretreatment with 10 μM mdivi-1, followed by DAPI staining (blue). n = 4 experiments. Size bar = 2 μm. B. Quantitation of mitochondrial morphology. > 150 cells were imaged and scored, as in Fig. 2B, as having reticular (black), fragmented (white), or intermediate (gray) morphology, n = 3 experiments. C. WT cells were incubated in the absence or presence of 10 μM mdivi-1, followed by treatment with 5 or 10 μM CCCP for 1 h and anti-TOM20 immunolabeling and DAPI staining. n = 4 experiments (PDF 12479 kb)

Supplemental Fig. 3. Low Δ*ψ*_m_ causes decreased cell viability. A. Confocal imaging of WT, WT + 5 μM CCCP, and ρ^0^ cells grown in high glucose media (top) and glucose-free galactose media (bottom). Cells visualized by brightfield and DAPI (cyan). n = 3 experiments. Size bar = 10 μm (PDF 17851 kb)

## Abbreviations

CCCP: Carbonyl cyanide m-chlorophenyl hydrazine
DAPI: Diaminophenylindole
DMEM: Dulbecco’s Modified Eagle’s medium
DMSO: Dimethylsulfoxide
EtBr: Ethidium bromide
FBS: Fetal bovine serum
MEF: Mouse embryonic fibroblast
mtDNA: Mitochondrial DNA
OxPhos: Oxidative phosphorylation
PBS: Phosphate-buffered saline
TMRE: Tetramethyl rhodamine ester
WT: Wild type
Δ*ψ*_m_: Mitochondrial transmembrane potential
