# Supplementary figures and images for: A threshold of transmembrane potential is required for mitochondrial dynamic balance mediated by DRP1 and OMA1
Source: Cell Mol Life Sci. 2016 Nov 17;74(7):1347–63. doi: 10.1007/s00018-016-2421-9 (PMC5346411; doi:10.1007/s00018-016-2421-9)

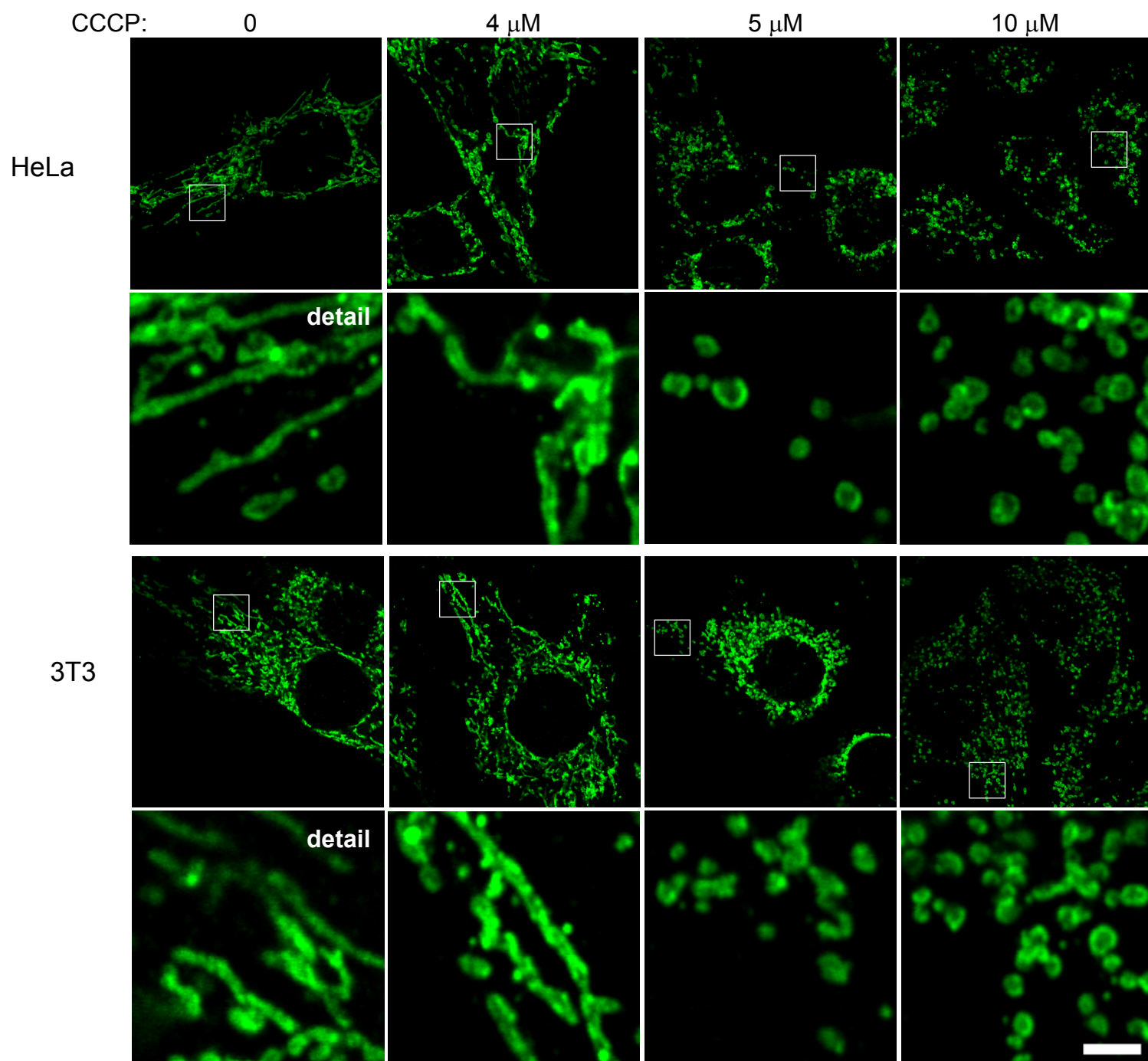

Supplement: Supplementary file 1 — Supplemental Fig. 1. Δψ m threshold of mitochondrial interconnection is a general phenomenon, and is reversible. Human HeLa and murine 3T3 cell lines were incubated in the absence or presence of CCCP at the indicated concentrations and visualized by anti-TOM20 immunolabeling. n = 3 experiments. Size bar = 2 μm (PDF 5764 kb) [file 18_2016_2421_MOESM1_ESM.pdf]

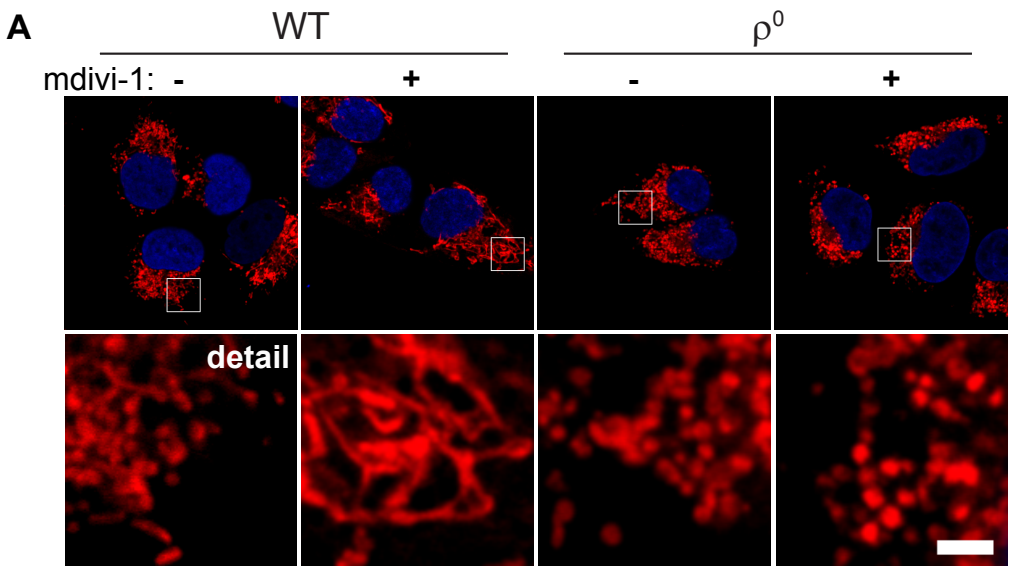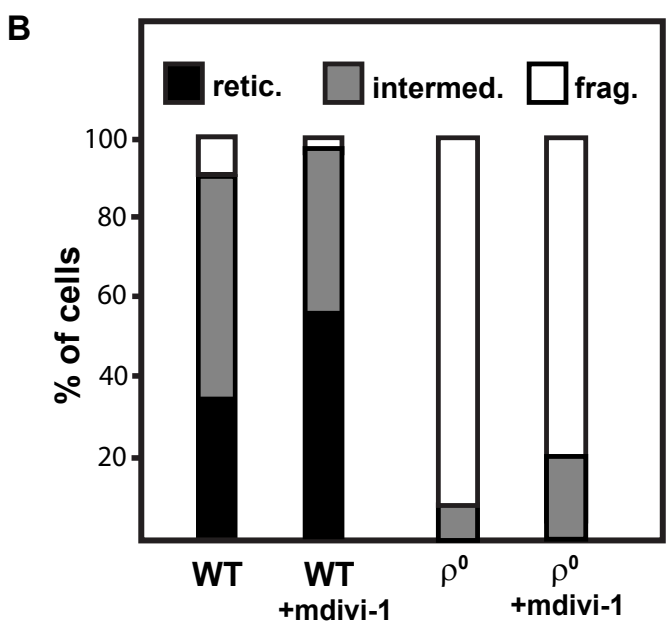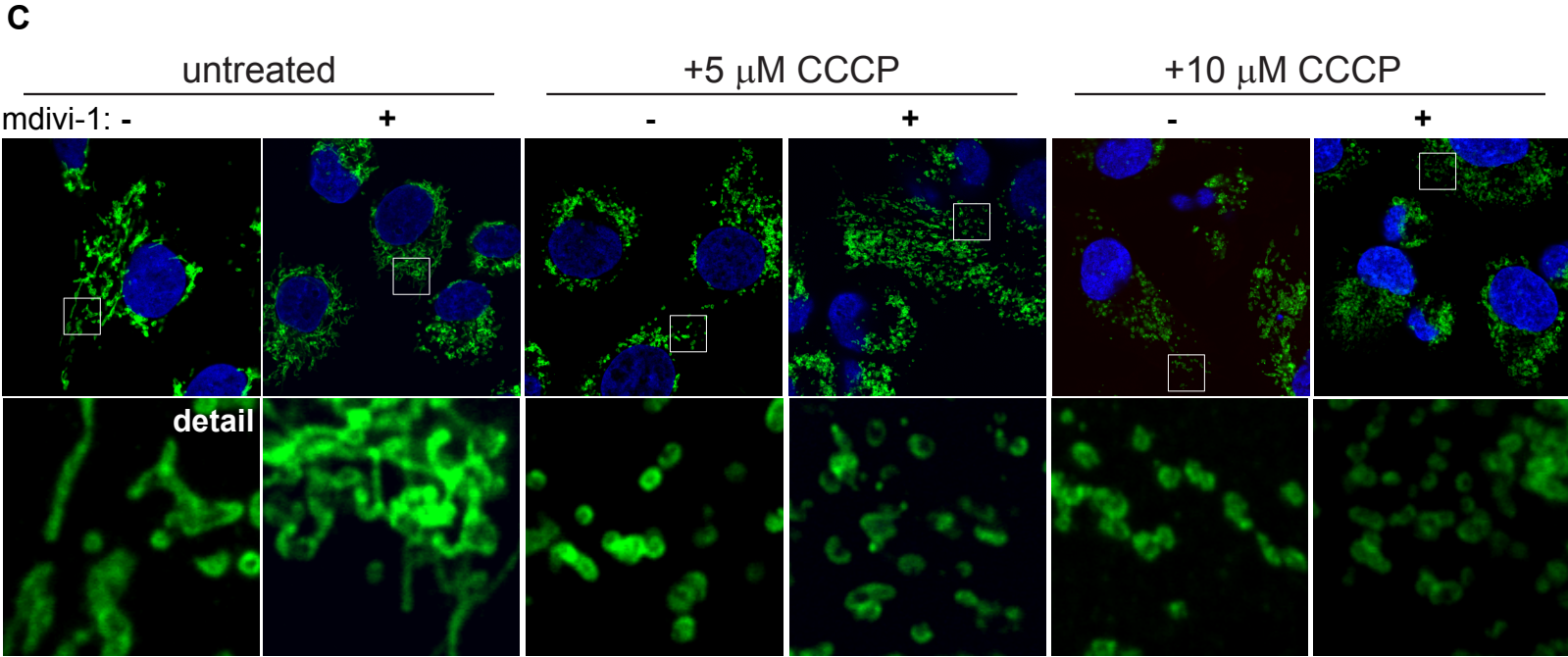

Supplement: Supplementary file 2 — Supplemental Fig. 2. Mdivi-1 does not increase fusion in cells with low Δψ m. A. WT and ρ0 cells were stained with MitoTracker (red) without (-) or with (+) overnight pretreatment with 10 μM mdivi-1, followed by DAPI staining (blue). n = 4 experiments. Size bar = 2 μm. B. Quantitation of mitochondrial morphology. > 150 cells were imaged and scored, as in Fig. 2B, as having reticular (black), fragmented (white), or intermediate (gray) morphology, n = 3 experiments. C. WT cells were incubated in the absence or presence of 10 μM mdivi-1, followed by treatment with 5 or 10 μM CCCP for 1 h and anti-TOM20 immunolabeling and DAPI staining. n = 4 experiments (PDF 12479 kb) [file 18_2016_2421_MOESM2_ESM.pdf]

**WT**

**WT+5  $\mu$ M CCCP**

**$\rho^0$**

**+glucose**

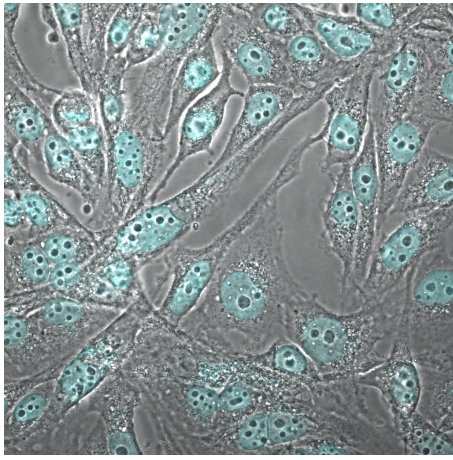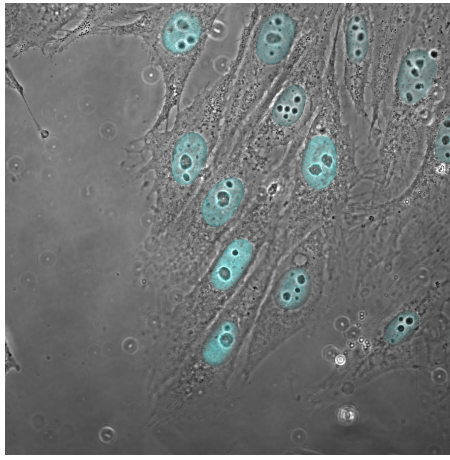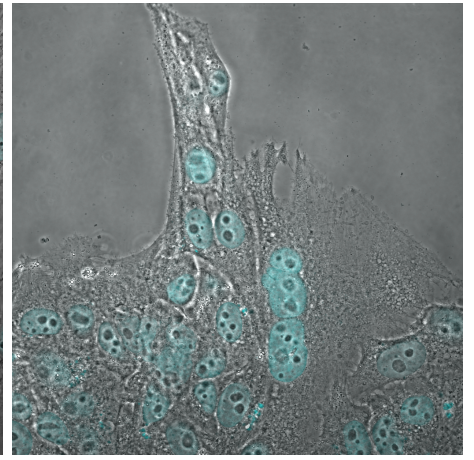

**-glucose  
+galactose**

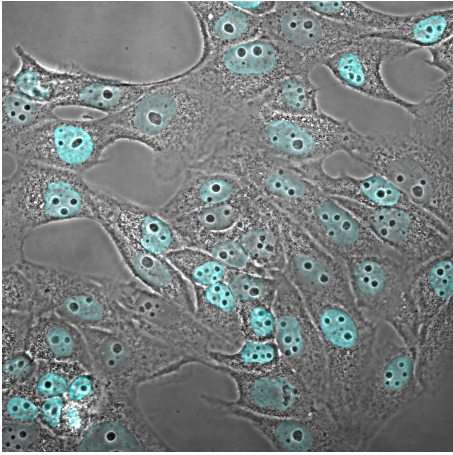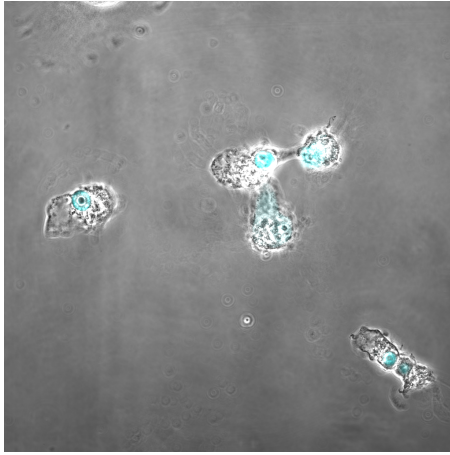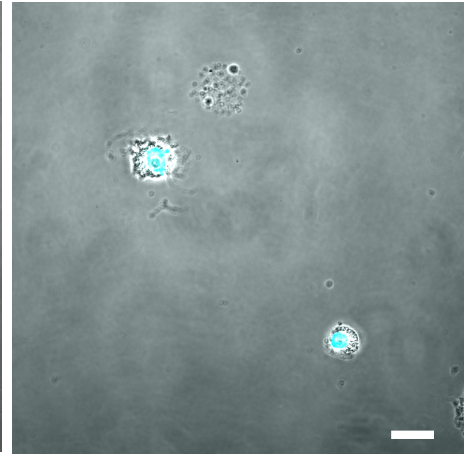

Supplement: Supplementary file 3 — Supplemental Fig. 3. Low Δψ m causes decreased cell viability. A. Confocal imaging of WT, WT + 5 μM CCCP, and ρ0 cells grown in high glucose media (top) and glucose-free galactose media (bottom). Cells visualized by brightfield and DAPI (cyan). n = 3 experiments. Size bar = 10 μm (PDF 17851 kb) [file 18_2016_2421_MOESM3_ESM.pdf]
